# Cardiorenal Protective Effect of Costunolide against Doxorubicin-Induced Toxicity in Rats by Modulating Oxidative Stress, Inflammation and Apoptosis

**DOI:** 10.3390/molecules27072122

**Published:** 2022-03-25

**Authors:** Wen Xing, Chaoling Wen, Deguo Wang, Hui Shao, Chunhong Liu, Chunling He, Opeyemi Joshua Olatunji

**Affiliations:** 1 Department of Gerontology, Wannan Medical College Affiliated Yijishan Hospital, Wuhu 241001, China; xingwen2004@sina.com (W.X.); wangdeguo@wnmc.edu.cn (D.W.); 2 Anhui Traditional Chinese Medicine College, Wuhu 241001, China; wenchaoling866@163.com; 3 Department of Clinical Laboratory, East China Normal University Affiliated Wuhu Hospital, Wuhu 241001, China; whshaohui@163.com; 4 The Second Peoples Hospital of Wuhu City, Wuhu 241001, China; 18155317015@163.com; 5 Department of Endocrinology, Wannan Medical College Affiliated Yijishan Hospital, Wuhu 241001, China; 6 Traditional Thai Medical Research and Innovation Center, Faculty of Traditional Thai Medicine, Prince of Songkla University, Hat Yai 90110, Thailand

**Keywords:** costunolide, cardiorenal protection, doxorubicin, oxidative stress, antioxidant

## Abstract

Doxorubicin (DXB) is one of the most commonly used anticancer agents for treating solid and hematological malignancies; however, DXB-induced cardiorenal toxicity presents a limiting factor to its clinical usefulness in cancer patients. Costunolide (COST) is a naturally occurring sesquiterpene lactone with excellent anti-inflammatory, antioxidant and antiapoptotic properties. This study evaluated the effect of COST on DXB-induced cardiorenal toxicity in rats. Rats were orally treated with COST for 4 weeks and received weekly 5 mg/kg doses of DXB for three weeks. Cardiorenal biochemical biomarkers, lipid profile, oxidative stress, inflammatory cytokines, histological and immunohistochemical analyses were evaluated. DXB-treated rats displayed significantly increased levels of lipid profiles, markers of cardiorenal dysfunction (aspartate aminotransferase, creatine kinase, lactate dehydrogenase, troponin T, blood urea nitrogen, uric acid and creatinine). In addition, DXB markedly upregulated cardiorenal malondialdehyde, tumor necrosis factor-α, interleukin-1β, interleukin-6 levels and decreased glutathione, superoxide dismutase and catalase activities. COST treatment significantly attenuated the aforementioned alterations induced by DXB. Furthermore, histopathological and immunohistochemical analyses revealed that COST ameliorated the histopathological features and reduced p53 and myeloperoxidase expression in the treated rats. These results suggest that COST exhibits cardiorenal protective effects against DXB-induced injury presumably via suppression of oxidative stress, inflammation and apoptosis.

## 1. Introduction

Doxorubicin (DXB), an anthracycline anticancer agent represents one of the most commonly used anticancer drugs in clinical practice for treating several hematological and solid malignancies [1,2]. Despite its clinical usefulness, DXB exerts significant cardiorenal toxicity upon continuous usage which could be life-threatening [1,3]. DXB-induced cardiotoxicity and nephrotoxicity have been extensively linked to the generation of reactive oxygen species, oxidative stress and deception of cellular antioxidant machineries leading to oxidative damage of renal and cardiac cellular structures [4,5,6].

DXB-induced cardiac toxicity is generally typified by high concentrations of lactate dehydrogenase, creatine kinase as well as cardiac morphological and functional alterations resulting in cardiomyopathy and heart failure [7,8,9], while DXB-induced nephrotoxicity is characterized by progressive loss of kidney structure and function, increased glomerular capillary permeability and tubular atrophy resulting in nephropathy [2,6]. 

As extensively illustrated in numerous reports, DXB-induced toxicity is primarily facilitated by the accumulation of ROS and oxidative stress, which consequently results in the stimulation of several other pathways related to cellular apoptosis and inflammation that can be lethal to several organs in the body [6]. As a means of mitigating chemotherapy-induced toxicity, antioxidant therapies have been recommended as a preventive approach for DOX-induced toxicities [10].

**Figure 1:**
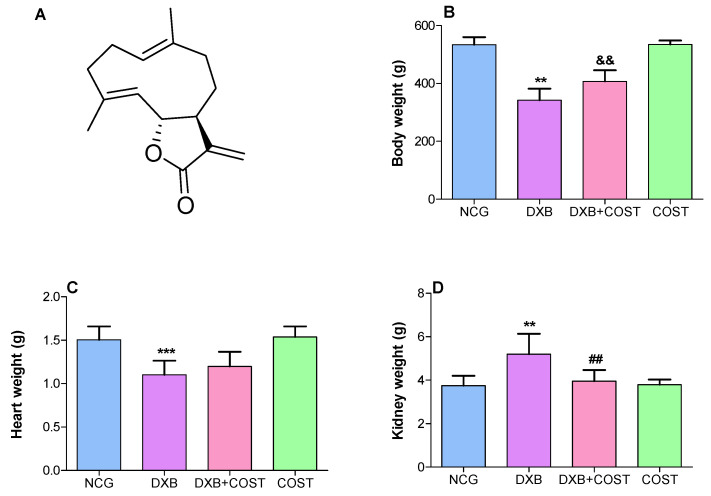

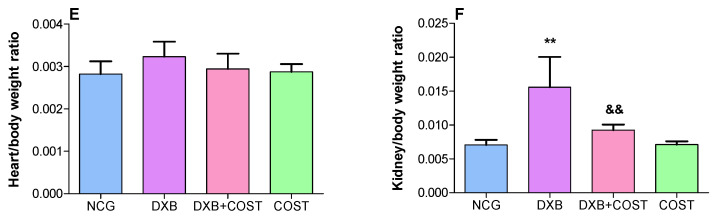
(**A**): Chemical structure of costunolide. Effect of costunolide on: (**B**): body weight gain, (**C**): heart weight, (**D**): kidney weight, (**E**): heart/body weight ratio, (**F**): kidney/body weight ratio. ** Statistically significant (*p* < 0.01) when compared with NCG and COST groups. && Statistically significant (*p* < 0.01) compared with DXB group. *** Statistically significant (*p* < 0.05) compared with NCG and COST groups. ## Statistically significant (*p* < 0.05) compared with DXB group.

Costunolide (COST; Figure 1A) is a naturally occurring sesquiterpene lactone isolated from several Compositae plants. Several notable biological activities have been attributed to COST, notably its anticancer efficacy against bone, breast and blood cancer [11,12]. COST was also reported to show several other bioactivities including antioxidant, anti-inflammatory, antiulcer, antidiabetic and neuroprotective properties [11,13,14,15]. However, there are no reports on the protective effects of COST on DXB-induced toxicity, including cardiotoxicity and nephrotoxicity. Due to the reported antioxidant and anti-inflammatory potentials of COST, this present study delineated the protective effects of COST against DXB-elicited oxidative cardiac and renal damage in rats. The effect of COST on cardiorenal antioxidant activities, inflammatory mediators, biochemical, histoarchitecture and immunohistochemical alterations in DXB-induced cardiorenal toxicity was evaluated.

## 2. Results

### 2.1. Effect of COST on Body, Heart and Kidney Weights

As shown in Figure 1, the results indicated a significant decrease in the body weight of DXB control rats compared to both HCG and COST treated rats. Whereas in the COST + DXB-treated rats, there was a conspicuous increase in body weight compared to the DXB rats. Furthermore, treatment of DXB-induced rats with COST significantly improved cardiac and renal weights, as well as attenuated renal/body weight ratio compared to the DXB group (Figure 1). 

### 2.2. Effect of COST on Cardiorenal Function Markers 

**Figure 2:**
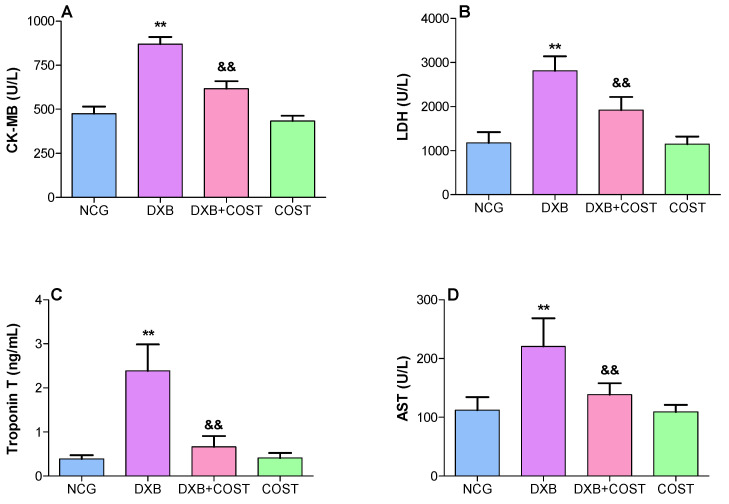

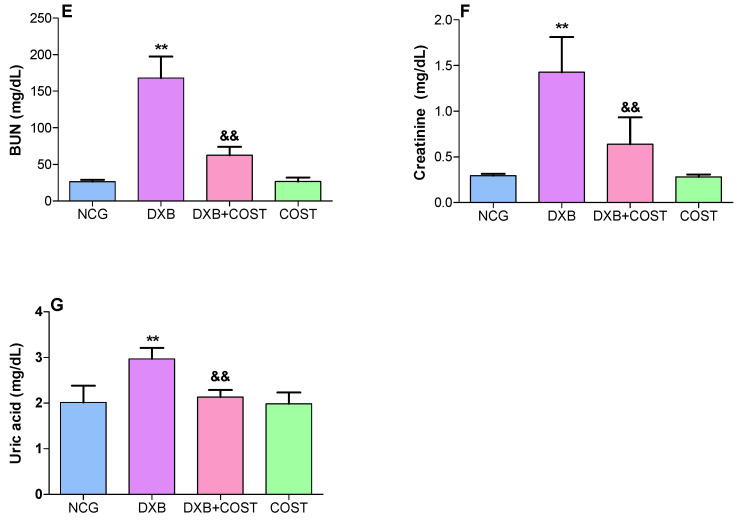
Effect of costunolide on: (**A**): CK-MB, (**B**): LDH, (**C**): Troponin T, (**D**): AST, (**E**): BUN, (**F**): creatinine, (**G**): uric acid. ** Statistically significant (*p* < 0.01) compared with NCG and COST groups. && Statistically significant (*p* < 0.01) compared with DXB group.

The results indicated that the administration of DXB significantly increased the serum levels of LDH, TnT, AST, CK-MB, BUN, uric acid and Scr compared to NCG and COST rats (Figure 2). In contrast, treatment of rats with COST significantly attenuated DXB-induced toxicity by markedly reducing the serum concentrations of these cardiorenal biomarkers compared to the DXB-treated rats (Figure 2).

### 2.3. Effects of COST on Serum Lipid Profiles 

**Figure 3:**
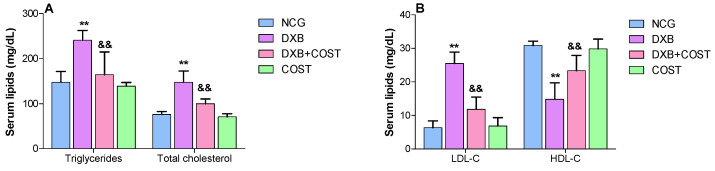
Effect of costunolide on: (**A**): serum triglycerides and total cholesterol, (**B**): serum LDL-C and HDL-C. ** Statistically significant (*p* < 0.01) compared with NCG and COST groups. && Statistically significant (*p* < 0.01) compared with DXB group.

As elucidated in Figure 3, DXB-treated rats showed notable disruptions in serum lipid profiles as indicated by worthy increases in the levels of TG, TC and LDL-C accompanied by significant decline in the serum HDL level when compared to NCG and COST rats. Contrariwise, treatment with COST substantially reduced TG, TC and LDL-C and simultaneously increased HDL levels compared to the DXB group (Figure 3). 

### 2.4. Effect of COST Treatment on Cardiorenal Antioxidants Activities 

**Figure 4:**
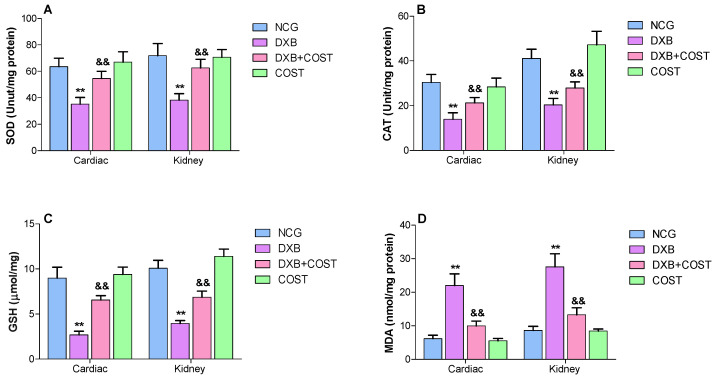
Effect of costunolide on: (**A**): SOD, (**B**): CAT, (**C**): GSH, (**D**): MDA. ** Statistically significant (*p* < 0.01) compared with NCG and COST groups. && Statistically significant (*p* < 0.01) compared with DXB group.

As indicated in Figure 4A–C, treatment with DXB led to marked diminution in cardiorenal antioxidant activities (GSH, SOD and CAT), while DXB administration also enhanced cardiorenal lipid peroxidation (MDA) levels compared to NCG and COST rats (Figure 4D). Whereas the protective effect of COST in reducing oxidative cardiorenal damage was evident in the restoration of cardiorenal antioxidant levels, the activities of SOD, GSH and CAT were significantly restored to levels similar to the NCG and COST groups. Meanwhile, MDA levels were markedly decreased in the COST + DOX treated group in comparison to the DXB control group (Figure 4).

### 2.5. Effect of COST Treatment on Cardiorenal Histopathological Analysis 

**Figure 5:**
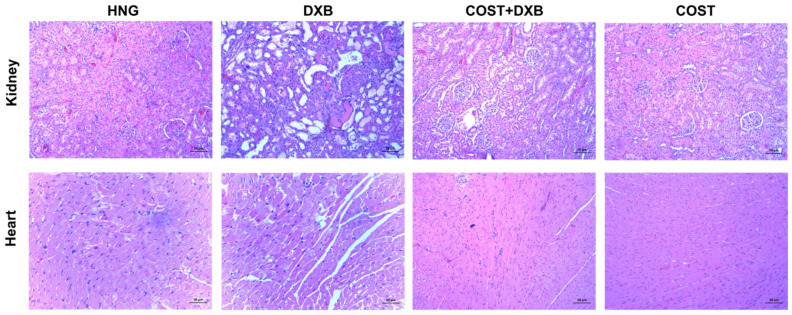
H&E stained photomicrography of the kidney and cardiac tissues from all tested groups. Kidney: red arrows; glomerular atrophy, black arrow; infiltration of inflammatory cells, green arrow; disintegration of renal tubules. Heart: blue arrow; inflammatory cells, brown arrows; degeneration of myofibrils (200×, 50 µm).

The representative histopathological images of the cardiorenal tissues are shown in Figure 5. The cardiac tissue of the DXB group showed histopathological alteration consistent with inflammatory cell infiltration and cardiac fiber necrosis, which were clearly absent in the NCG and COST group. Treatment with COST evidently restored the altered cardiac histoarchitecture compared to the untreated DXB group.

The representative microscopic sections of the kidney tissues of the NCG and COST groups revealed normal kidney morphology of the cortical tubules and the glomerulus without any evident alterations. In contrast, the DXB-treated group showed significant deteriorative changes including infiltration of inflammatory cells, glomerular atrophy, dilation of the Bowmans capsules as well as disintegration of renal tubules. Whereas in the COST treated group, significant alleviation of these alterations as well as significant preservation of the integrity of the renal architecture was observed when compared to the DXB alone treated group (Figure 5).

### 2.6. Effects of COST Treatment of Cardiorenal Proinflammatory Cytokines

**Figure 6:**
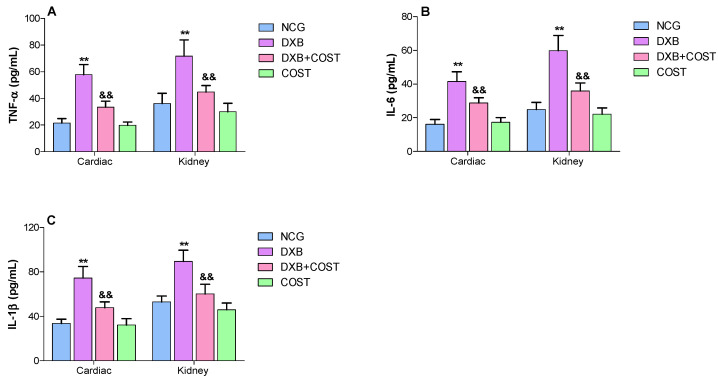
Effect of costunolide on: (**A**): TNF-α, (**B**): IL-6, (**C**): IL-1β. ** Statistically significant (*p* < 0.01) compared with NCG and COST groups. && Statistically significant (*p* < 0.01) compared with DXB group.

In the DXB alone treated group, significant increases in cardiorenal levels of proinflammatory cytokines viz TNF-α, IL-6 and IL-1β were observed compared to the NCG and COST groups (Figure 6A–C). Treatment with COST induced marked decreases in cardiorenal concentrations of TNF-α, IL-6 and IL-1β compared to DXB group (Figure 6A–C).

### 2.7. Effects of COST Treatment of Cardiorenal p53 and Myeloperoxidase

**Figure 7:**
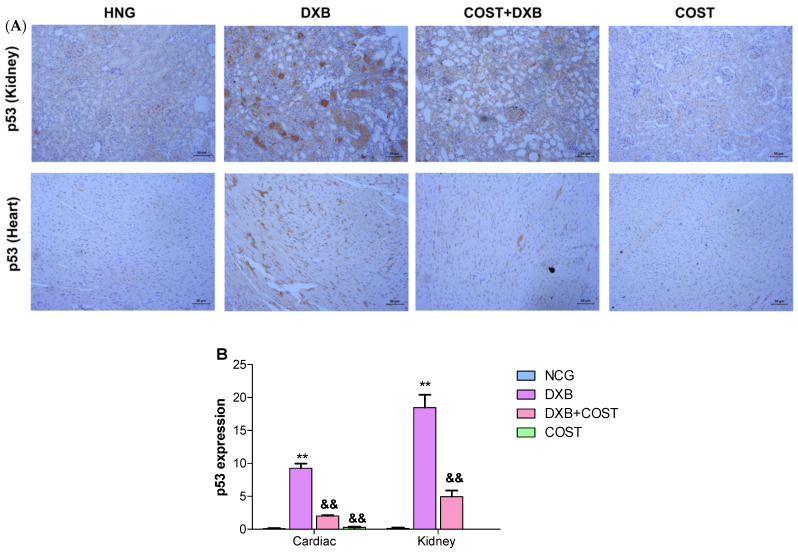
(**A**): Photomicrography of kidney and heart tissues showing expression of p53 in all experimental groups, (**B**): quantification of p53 immunohistochemical expression in kidney and heart tissues. ** Statistically significant (*p* < 0.01) compared with NCG and COST groups. && Statistically significant (*p* < 0.01) compared with DXB group.

**Figure 8:**
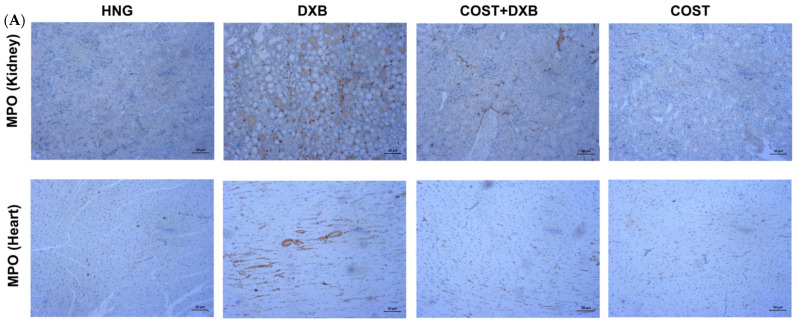

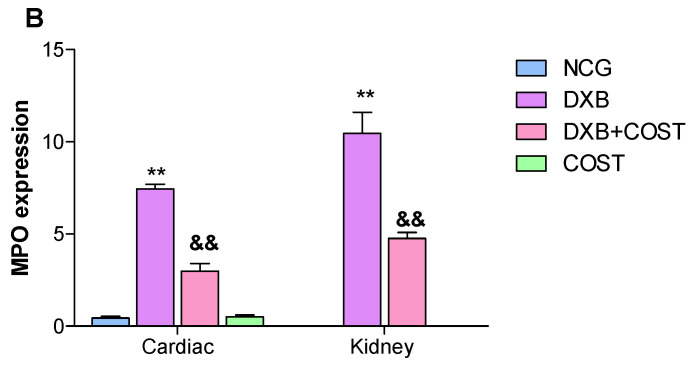
(**A**): Photomicrography of kidney and heart tissues showing expression of MPO in all experimental groups, (**B**): quantification of MPO immunohistochemical expression in kidney and heart tissues. ** Statistically significant (*p* < 0.01) compared with NCG and COST groups. && Statistically significant (*p* < 0.01) compared with DXB group.

The effect of COST on p53 and MPO expression was assessed in cardiorenal tissues using immunohistochemical analysis. Compared with the HCG and COST group, the DXB group showed significantly intense positive staining for p53 (Figure 7) and MPO (Figure 8) in both cardiac and renal tissues. On the contrary, treatment of DXB-injected rats with COST markedly reduced the expression of p53 and MPO compared to the DXB untreated group (Figure 7A,B and Figure 8A,B). 

## 3. Discussion

DXB represents a prominent antitumor agent for the treatment of a wide range of malignancies [16]; however, prolonged administration of DXB has prevailing adverse effects including cardiotoxicity, nephrotoxicity and neurotoxicity. The multi-organ injury associated with DXB has been widely linked to the increased production of oxidative radicals, oxidative stress and depleted cellular antioxidant availability, resulting in oxidative tissue damage [17]. Prevailing evidence from numerous studies have illustrated DXB-induced oxidative injury in the heart, kidney, brain and testes [6,18,19,20]. Therefore, exploring therapies that can prevent this oxidative multi-organ damage exerted by DXB in cancer patients is warranted. This present study investigated the protective effects of COST against DXB-induced cardiorenal toxicity in rats. The results obtained clearly showed that the administration of multiple doses of DXB induced oxidative damage, resulting in cardiorenal, biochemical and pathological alterations, whereas treatment with COST significantly ameliorated the oxidative induced damage.

In the current study, DXB-treated animals showed significantly increased serum levels of BUN, Scr, uric acid, LDH, TnT, AST and CK-MB. The increase in the concentrations of these serum cardiorenal biomarkers corresponds to compromised cardiorenal architecture including myocardial and tubular injury. The disruption in cellular membranes of the cardiac and renal tissues ultimately results in the release of these enzymes into the blood [7,21,22]. Furthermore, increases in these serum cardiorenal biomarkers following DXB administration corresponded with significant histopathological injury as revealed by myocardial and renal tubule degeneration, infiltration of inflammatory cells, as well as disarrangement of cardiorenal tissues. These results corroborated those of previous studies [22,23]. Contrariwise, the results obtained indicated that COST significantly improved the levels of these serum cardiorenal biomarkers as well as ameliorated histopathological damage accrued on cardiac and renal tissues by DXB.

Several studies have shown the prominent role of lipids in cardiovascular diseases and the role of DXB-induced hyperlipidemia has been well documented [24,25]. DXB interferes with the metabolism and synthesis of lipids resulting in high serum and cardiac lipid profiles [9,16]. In addition, it was reported that treatments exhibiting antihyperlipidemic effects can decrease DXB-induced cardiotoxicity [26,27]. The administration of DXB was associated with significant increases in serum TG, TC and LDL-C, while HDL was markedly reduced, corroborating the findings of previous studies [24,25]. COST significantly ameliorated DOX-induced hyperlipidemia in the treated rats. 

As earlier stated, the ability of DXB to instigate ROS and oxidative stress is the most prevailing factor associated with cardiorenal toxicity associated with DXB. A growing body of evidences has proposed the implication of ROS-prompted oxidative injury in multiple pathways involved in DXB-induced toxicity [4]. It is well known that ROS stimulates the generation of reactive radicals that can interact with superoxide radicals leading to the generation of highly toxic peroxynitrite species, resulting in damage to proteins and macromolecules forming the basis of cardiorenal damage [6]. The results from this study revealed DXB administration instigated cardiorenal oxidative stress, as displayed by marked regression in GSH, SOD and CAT activities and elevated MDA levels. The decrease in these cellular antioxidative enzyme activities was in line with several earlier studies suggesting the inability of the renal and cardiac tissues to scavenge the increase in toxic reactive oxygen species and lipid peroxides [7,28,29]. Previous studies have illustrated the antioxidant prowess of costunolide in several models of toxicity. Mao et al. [17], reported the protective effects of COST against acute liver injury induced by D-galactosamine and LPS due to its antioxidative activity. Similarly, costunolide also showed protective effects against ethanol-induced gastric ulcer partly by increasing SOD activity and decreasing MDA levels [14]. In this study, COST treatment significantly increased SOD, GSH and CAT activities in the heart and kidneys of DXB-induced rats.

Moreover, increased generation of ROS and oxidative stress can exacerbate the inflammatory cascade, leading to cardiorenal dysfunction. Another critical factor involved in DXB-induced toxicity is inflammation, which involves the degradation of NF-κB protein and subsequent enhancement of proinflammatory cytokine generation [30]. The involvement of several proinflammatory cytokines particularly IL-6, IL-1β and TNF-α in DXB-induced cardiorenal toxicity was extensively highlighted by previous studies [31,32]. Oxidative-induced translocation and modulation of NF-κB to the nucleus stimulates the transcription of proinflammatory cytokine genes [7,30,33,34]. Moreover, a previous study indicated that DXB significantly activated NF-κB and IKKα, which subsequently modulated the levels of TNF-α, IL-1β and IL-6 [30]. The overexpression of these proinflammatory cytokines together with other inflammatory related mediators results in severe consequences. In addition, DXB administration also significantly increased the stain intensity of MPO in cardiorenal tissues suggesting neutrophil infiltration. It is interesting to note that the anti-inflammatory effects of COST have been widely reported in many studies. Xie et al. indicated the COST significantly suppressed IL-1β, IL-6, TNF-α and NF-κB in dextran sulfate treated mice [35]. In another study, COST attenuated lipoteichoic acid-induced acute lung injury by reducing TNF-α, IL-6 and MAPK inflammatory pathway [36]. In the present study, treatment of DXB-administered rats with COST significantly attenuated cardiorenal proinflammatory cytokine levels (TNF-α, IL-1β and IL-6), revealing another facet of the cardioprotective properties of COST. The results obtained in this study corroborated those of preceding studies.

Apoptosis was also identified as one of the several mechanisms associated with DXB-induced cardiorenal injury. Several apoptotic proteins were shown to be critically upregulated in cardiorenal tissue toxicity following DXB intoxication [3,4]. Exposure to DXB significantly increased cardiorenal expression of p53, an important apoptotic mediator. Earlier studies demonstrated that DXB toxicity induces up-regulation in p53 protein in the kidney, testes and cardiac tissues [37,38,39,40]. However, COST treatment down-regulated cardiorenal apoptosis by inhibiting increases in p53 intensity, thus showing protective effects against DXB-mediated toxicity.

## 4. Materials and Methods

### 4.1. Chemicals and Reagents 

Costunolide was gratefully provided by Professor Jian Tang (Bozhou University, China). DXB was the product of Fresenius Kabi Oncology Ltd., Haryana, India. The kits used for assaying superoxide dismutase (SOD), malondialdehyde (MDA), catalase (CAT) and glutathione (GSH) were products of Jiancheng Biotechnology Science, Nanjing, China. ELISA kits for the estimation of tumor necrosis factor (TNF)-α, interleukin (IL)-1β and IL-6 were procured from Abcam (Cambridge, UK). 

### 4.2. Animals and Experimental Treatment 

A total of 24 Sprague–Dawley rats (aged 8 weeks) of specific pathogen-free grade were accommodated in cages with six rats each in an air-conditioned room with the temperature set at 22 ± 2 °C, and a 12 h/12 h light/darkness diurnal cycle. The animals had unrestricted access to food and water and were acclimatized for 7 days prior to the commencement of the experiment. The experimental protocol was approved by the Ethics Committee of Wannan Medical College Affiliated Yijishan Hospital, China (WNYXYYJSYY-2021-1008). After the period of acclimatization, the rats were unbiasedly allotted into four groups as follows:

Group 1: Healthy control group (HCG), received 5% DMSO orally for 4 weeks
Group 2: DXB control group (DXB), received 5% DMSO orally for 4 weeks
Group 3: COST + DXB group, received COST (50 mg/kg) orally for 4 weeks.
Group 4: COST group (COST), received COST (50 mg/kg body weight) orally for 4 weeks.

The rats in the DXB and COST + DXB groups were intraperitoneally administered with 5 mg/kg of DXB once a week for three weeks (total dose: 15 mg/kg) from the second week to the fourth week of treatment to induce cardiorenal toxicity, while the rats in the HCG and COST groups were also intraperitoneally administered with the same volume of normal saline instead of DXB during the same period. The doses of COST and DXB used in this study were adopted from previous studies [6,41,42]. After the treatment, the animals were fasted overnight, anesthetized with thiopental sodium and blood samples were collected for serum biochemical analysis. The cardiac and renal tissues were harvested, weighed and preserved at −80 °C for biochemical assays. Fresh tissue samples were also preserved in 10% buffered formalin solution for histopathological and immunohistochemical analyses.

### 4.3. Biochemical Assays

The blood samples were centrifuged, and the serum obtained was used for determining lactate dehydrogenase (LDH), troponin T (TnT), aspartate transaminase (AST), creatine kinase-MB (CK-MB), blood urea nitrogen (BUN), uric acid, creatinine (Scr), lipids (total cholesterol (TC), triglycerides (TG), low density lipoprotein cholesterol (LDL-C) and high density lipoprotein (HDL)).

### 4.4. Antioxidant and Proinflammatory Cytokines Parameters 

The cardiorenal tissues were centrifuged in phosphate-buffered saline and then centrifuged at 12,000× *g* for 15 min at 4 °C. The tissue homogenate obtained after decanting was used for further assessing oxidative stress parameters (MDA, SOD, CAT and GSH) using biochemical kits. Moreover, the levels of TNF-α, IL-6 and IL-1β in the cardiac and renal tissue homogenates were assayed using ELISA kits following the manufacturer’s protocol.

### 4.5. Hematoxylin and Eosin Staining

Cardiorenal tissues fixed in 10% buffered formalin were dehydrated with alcohol, embedded in paraffin wax and cut into 5 µM sections. The tissues were further stained with hematoxylin and eosin. The stained sections were visualized under a light microscope.

### 4.6. Immunohistochemical Expression of p53 and Myeloperoxidase 

For immunohistochemistry analysis, the paraffin embedded tissue sections were rehydrated in xylene and then rehydrated by utilizing graded ethanol solutions. Following this, sections were immunostained with primary antibodies (anti-p53 and anti-MPO antibodies) at 4 °C overnight. Thereafter, the slides were washed with PBS and further incubated with secondary antibodies at 4 °C for 2 h. Sections were further treated with a solution of 3,3′-diaminobenzidine with 0.03% hydrogen peroxide for 10 min and counter stained with hematoxylin. The slides were visualized under a light microscope and immunohistochemical quantification was performed with image-J software. 

### 4.7. Statistical Analysis

All data were presented as mean ± standard deviation (SD) and analyzed using GraphPad Prism 5 software (San Diego, CA, USA). Statistical differences between groups were analyzed using one-way ANOVA with Bonferroni post-test. Statistical significance was set at *p* < 0.05.

## 5. Conclusions

In conclusion, the present study demonstrated that COST exerted cardiorenal protective effects by modulating antioxidant defense, reducing inflammation, apoptosis and preventing damage to cardiorenal structures. Since oxidative stress is considered to play a vital role in the pathophysiology of DXB-induced cardiorenal toxicity, and considering that COST significantly ameliorated oxidative stress and inflammatory biomarkers in DXB treated rats, it is therefore reasonable to hypothesize that the cardiorenal protective effects of COST may be largely mediated through its antioxidant activity and anti-inflammatory properties.

## Data Availability

The data sets used and/or analyzed in this study are available from the corresponding author on reasonable request.

## Sample Availability

Samples of the compounds are not available from the authors.
